# Challenging Global Waste Management – Bioremediation to Detoxify Asbestos

**DOI:** 10.3389/fenvs.2020.00020

**Published:** 2020-03-04

**Authors:** Shannon L. Wallis, Edward A. Emmett, Robyn Hardy, Brenda B. Casper, Dan J. Blanchon, Joseph R. Testa, Craig W. Menges, Cédric Gonneau, Douglas J. Jerolmack, Ali Seiphoori, Gregor Steinhorn, Terri-Ann Berry

**Affiliations:** 1 Engineering Pathway, Unitec Institute of Technology, Auckland, New Zealand,; 2 Perelman School of Medicine, Superfund Research Program, University of Pennsylvania, Philadelphia, PA, United States,; 3 Faculty of Arts and Design, University of Canberra, Canberra, ACT, Australia,; 4 Department of Biology, University of Pennsylvania, Philadelphia, PA, United States,; 5 School of Environmental and Animal Sciences, Unitec Institute of Technology, Auckland, New Zealand,; 6 Fox Chase Cancer Center, Philadelphia, PA, United States,; 7 Department of Earth and Environmental Science, University of Pennsylvania, Philadelphia, PA, United States,; 8 Research and Enterprise, Unitec Institute of Technology, Auckland, New Zealand

**Keywords:** asbestos, hazardous waste treatment, bioremediation, waste minimisation, carcinogenicity

## Abstract

As the 21st century uncovers ever-increasing volumes of asbestos and asbestos-contaminated waste, we need a new way to stop ‘grandfather’s problem’ from becoming that of our future generations. The production of inexpensive, mechanically strong, heat resistant building materials containing asbestos has inevitably led to its use in many public and residential buildings globally. It is therefore not surprising that since the asbestos boom in the 1970s, some 30 years later, the true extent of this hidden danger was exposed. Yet, this severely toxic material continues to be produced and used in some countries, and in others the disposal options for historic uses – generally landfill – are at best unwieldy and at worst insecure. We illustrate the global scale of the asbestos problem via three case studies which describe various removal and/or end disposal issues. These case studies from both industrialised and island nations demonstrate the potential for the generation of massive amounts of asbestos contaminated soil. In each case, the final outcome of the project was influenced by factors such as cost and land availability, both increasing issues, worldwide. The reduction in the generation of asbestos containing materials will not absolve us from the necessity of handling and disposal of contaminated land. Waste treatment which relies on physico-chemical processes is expensive and does not contribute to a circular model economy ideal. Although asbestos is a mineral substance, there are naturally occurring biological-mediated processes capable of degradation (such as bioweathering). Therefore, low energy options, such as bioremediation, for the treatment for asbestos contaminated soils are worth exploring. We outline evidence pointing to the ability of microbe and plant communities to remove from asbestos the iron that contributes to its carcinogenicity. Finally, we describe the potential for a novel concept of creating ecosystems over asbestos landfills (‘activated landfills’) that utilize nature’s chelating ability to degrade this toxic product effectively.

## INTRODUCTION

**FIGURE 1 |:**
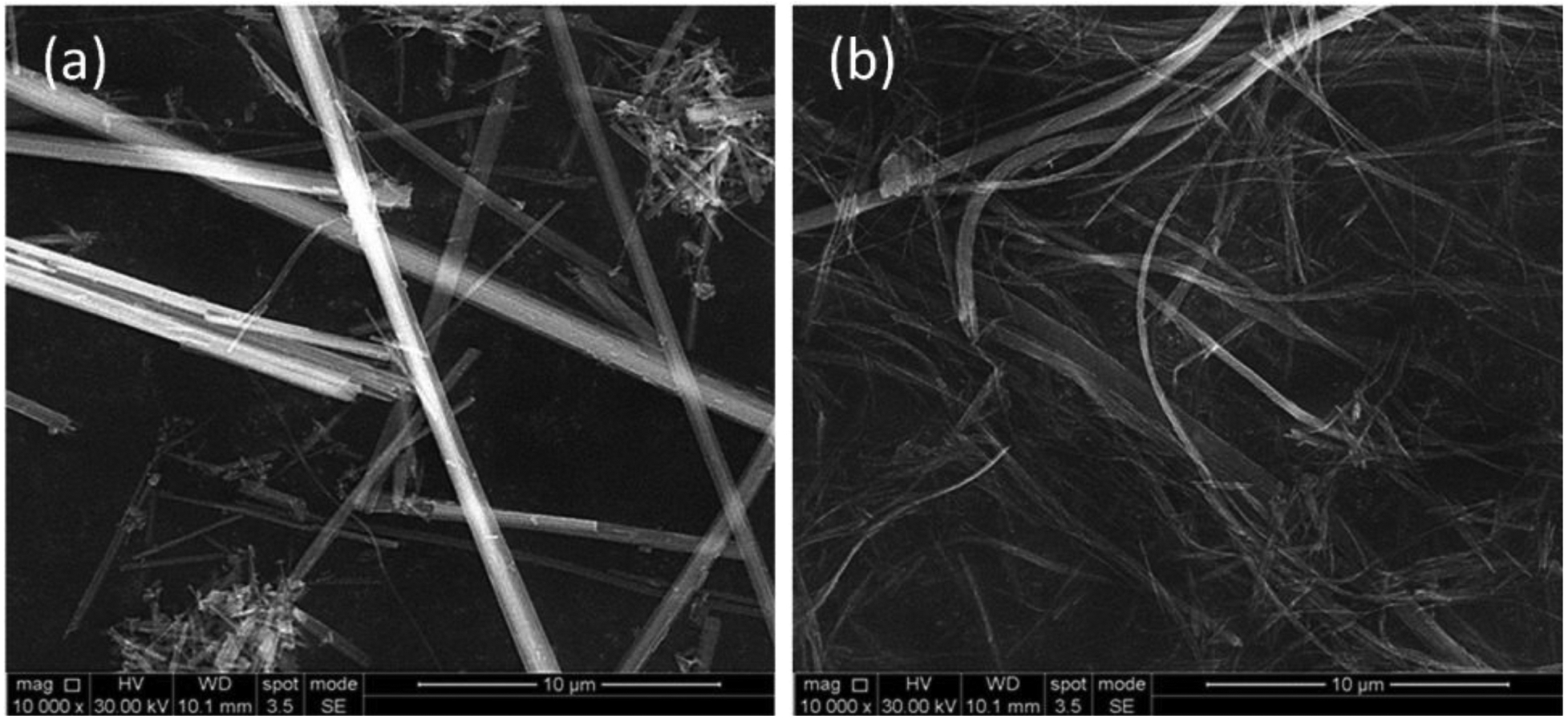
Scanning electron photomicrographs (SEM) of two members of amphibole and serpentine asbestos family, respectively (**a**) the crocidolite or blue asbestos (from Koegas, South Africa), and (**b**) the Canadian B chrysotile.

Asbestos is a term applied to six naturally occurring fibrous silicate-based minerals, for which there are two configurations based on chemistry and morphology: serpentine and amphibole; see Figure 1. Chrysotile (white asbestos) is derived from serpentine minerals and accounts for 95% of all the asbestos used in the 20th century and 100% of the asbestos used today (Virta, 2005). Of the amphibole minerals, the most commercially successful forms were amosite (also known as brown asbestos) and crocidolite (or blue asbestos) (LaDou et al., 2010). There are also other carcinogenic fibrous silicates, variously referred to as elongated mineral fibres (EMF) (Carlin et al., 2015) or asbestiform minerals, that have similar toxicity but are not classified as asbestos for regulatory purposes (Frank and Joshi, 2014). Asbestos’s valuable physico-chemical properties – resistance to heat and fire, insulation capability, chemical inertness and strength (Godish, 1989) – led to its widespread use last century and a production peak in the 1970s (Radetzki, 2010). Asbestos-containing materials (ACM) have been used in floor and ceiling tiles, surfacing materials, thermal insulation around pipes and boilers, wallboard, roofing material and many other applications (Godish, 1989; New Zealand Ministry for Education, 2015) and in more than 3,000 manufactured products (LaDou, 2004). Worldwide, some 200 million tonnes of asbestos have been mined and used in products since 1900 (Vogel, 2005; Haynes, 2010).

Its production has now tapered. Asbestos mining, importation and use is totally banned in 57 countries (International Ban Asbestos Secretariat, 2017). In the United States, the limited remaining uses are severely restricted, but prominent asbestos mining and/or use continues in Russia, China, India, and Kazakhstan (Leong et al., 2015). Current global production is estimated at around two million tonnes per annum (United States Geological Survey [USGS], 2013). Consequently, low- to middle-income countries, that require high industrial growth and often have poor environmental controls, continue to import and use asbestos. Safer alternatives exist in the form of artificial materials, but the tariffs some countries impose on these are barriers to discontinuing asbestos use (Frank and Joshi, 2014). Large quantities of asbestos remain as a legacy from past use in construction of many residential and commercial buildings (Ramazzini, 2010). Asbestos waste materials from demolition or removal are often disposed of in controlled landfills. While this practice may be effective over the short-term, it does not completely eliminate future fibre release and does not support sustainable land use or recycling (Spasiano and Pirozzi, 2017). Removal and disposal costs are considerable, and in New Zealand there is anecdotal evidence of communities burying asbestos-contaminated waste in unmarked, uncontrolled areas to avoid the costs.

Asbestos is rarely disposed of in its pure fibrous form but is commonly combined within a building material matrix, commonly concrete. Since this asbestos exists as part of a matrix, rather than as loose fibres, this greatly increases the volume to landfill. From usage to date, if we conservatively assume an average asbestos concentration of 5%, the total contaminated waste ultimately requiring disposal is on the order of 4 billion tonnes. As soil asbestos levels as low as 10 mg/kg is required by some legislation, disposal volumes of contaminated soil, plus contaminated waste, are likely to exceed this estimate (Government of Western Australia Department of Health, 2009). Deteriorating asbestos-containing building materials and continuing use of asbestos in some countries will only add to this burden, generating quantities of asbestos (both ACM and loose fibres in soils) that easily exceed the low acceptable limits (Table 1). Currently, no long-term unified approach addresses either the increasing waste volumes or the legacy of multiple sites of marked (or unmarked) contaminated land. Even materials with a very low asbestos content (*<*1% by weight), including contaminated soils, can generate hazardous levels of exposure when disturbed (Carlin et al., 2015).

This vast volume of ACM is therefore unwieldy. Its disposal using current methods raises the prospect of a ‘fourth wave’ of asbestos exposure and subsequent asbestos-related disease (ARD) arising from the ACM waste itself. The asbestos wave concept (Landrigan, 1992) proposes that the first wave arose from exposure to those who mined, milled and packaged asbestos; the second from the production and installation of asbestos-containing building materials and other products; and a third from exposure to asbestos in place, such as in buildings, which includes groups such as do-it-yourself home renovators (Olsen et al., 2011) and those who live with asbestos contaminants in their neighbourhood as in abandoned factory sites and ACM-contaminated waste sites (Emmett and Cakouros, 2017).

**FIGURE 2 |:**
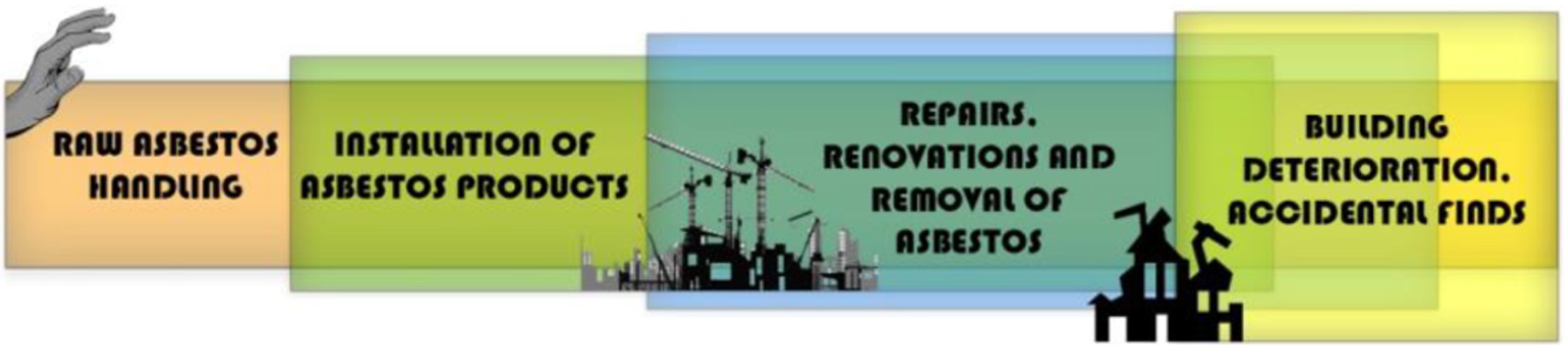
The four waves of asbestos-related diseases.

A fourth wave could arise from unrecognised, accidental exposure to asbestos in and around historic landfills and illegal deposits of contaminated waste (Figure 2). This risk might be increased in regions with insufficient record keeping, where relevant documents are lost due to political turmoil or conflict, or from undocumented ACM disposal. Furthermore, as demonstrated in our Cook Islands case study [see section “Case Study 2 – Asbestos-Contaminated Land in the Cook Islands (Unitec Institute of Technology)”], increasing demand for land availability over time may increase the likelihood of exposing buried ACM.

**FIGURE 3 |:**
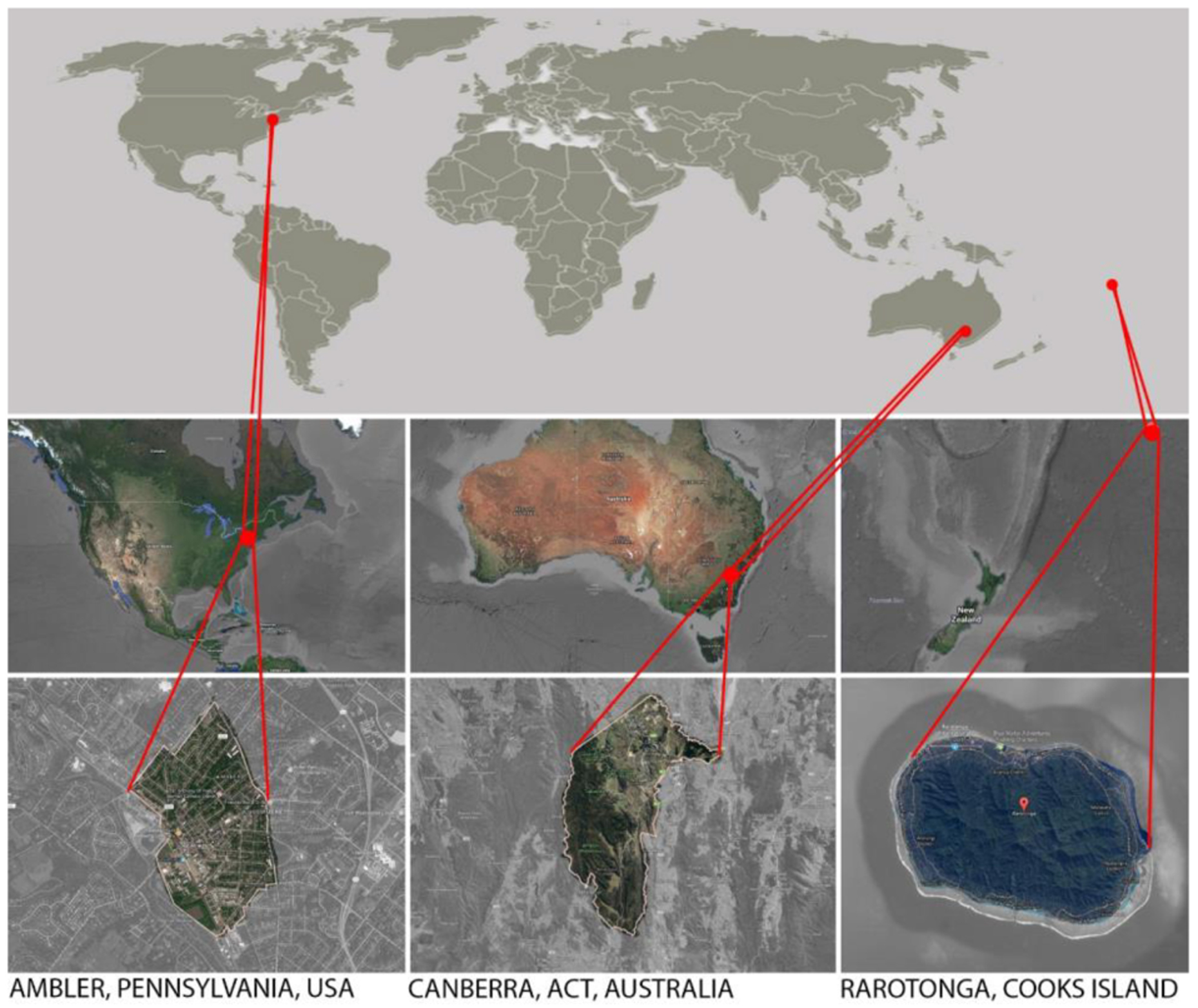
The locations of case studies involving asbestos removal and disposal in United States, Australia, and the Cook Islands.

In this paper, we present case studies describing contemporary asbestos removal and disposal practices in three different countries (Figure 3), which highlight the increasing landfill burdens of asbestos-contaminated construction waste and soils. We investigate practicalities and financial implications associated with the safe removal, transportation and disposal of asbestos contaminated soils and materials. We consider the sustainability and longevity of disposal options other than landfill disposal which include solutions based on waste detention, such as on-site capping. Our case studies demonstrate that, in particular, the disposal of asbestos contaminated soils remain an unsolved problem of significant magnitude, globally. These soils may contain very low levels of asbestos and yet contribute a large volume of waste to hazardous landfill. Physico-chemical treatment options are unlikely to be applied as sustainable solutions for this waste stream but there may be opportunities for biological treatment systems. We discuss why bioremediation could be an effective and, ultimately, safer alternative to landfill disposal and outline planned trials for bioremediation of asbestos-contaminated soils.

## ASBESTOS TOXICITY

It is difficult to overstate the impact of asbestos on environmental and occupational health. All forms of asbestos can cause all of the ARDs, with no safe form or exposure level (International Agency for Research on Cancer [IARC] and World Health Organisation [WHO], 2012). This includes asbestiform minerals, such as erionite and antigorite and all forms of asbestos found as a natural contaminant of other minerals, e.g., talc, vermiculite and feldspar. The World Health Organization currently estimates that asbestos exposure causes more than half the deaths from occupational cancers worldwide.

The most important ARD are malignant mesothelioma (MM), lung cancer and asbestosis. For all of these, the latency period from the time of first inhalation of asbestos to detectable disease is characteristically long; for mesothelioma, the risk of disease plateaus around 50 years after first exposure (Reid et al., 2014). Asbestos is a major cause of MM, so the disease is an important marker of asbestos exposure (although recently, exposure to other asbestiform minerals has also been linked to MM cases (Carbone and Yang, 2012). Asbestos acts as a carcinogen in lung carcinoma, and the combination of cigarette smoking and asbestos exposure greatly increases the risk of lung cancer (Barrett et al., 1989). Asbestosis is a respiratory disease marked by inflammation and scarring of the lungs that restricts lung expansion (Carbone et al., 2011).

Asbestos inhalation causes inflammation and consequent carcinogenic activity and it is the iron at the surface of the asbestos fibre that may be responsible (Pollastri et al., 2015; Balamurugan et al., 2018). Iron is an element that bioremediation can potentially diminish. Active iron at the surface of the fibres induces hydrogen peroxide (H_2_O_2_) production from immune cells; quantification of the release of H_2_O_2_ and other reactive oxygen species (ROS) continues to be a focus for investigation (Balamurugan et al., 2018). In the case of asbestos, fibre surface area is a better predictor of inflammatory response than fibre mass or number (Duncan et al., 2014). Furthermore, a fibre that does not contain iron does not induce ROS or subsequent cellular damage. Iron is capable of binding to DNA within pulmonary cells. Its chemical reduction promotes the formation of the highly reactive hydroxyl radical (HO·) and this could occur in the immediate vicinity of the DNA, thus promoting carcinogenesis.

Even a very small amount of iron induces radical activity (Pollastri et al., 2015), for which the iron’s position within the fibre is important. The iron must be available at the fibres surface to be in contact with the H_2_O_2_ released during persistent inflammatory activity. Progressive dissolution of the fibre can make bulk iron available at the surface. For example, chrysotile (mean fibre size, 0.1 μm ϕ) is not iron-rich, but it has a predicted fibre dissolution rate greater than that of either amosite or crocidolite. Therefore, despite its low iron content, the iron is more available; the ability of chrysotile fibres to generate available surface iron-related HO· is predicted to be as high as for more iron-rich crocidolite fibres (Pollastri et al., 2015).

Asbestos-induced disease, therefore, depends on factors that include: asbestos fibre size, surface activity, ability to generate ROS, bio persistence, iron content and iron-bodies formation (Pollastri et al., 2015). The presence and speciation (both oxidation state and coordination environment) of iron are important factors affecting toxicity (Pollastri et al., 2015). Chrysotile asbestos’s carcinogenicity can be reduced by removing iron with organic acids (Chao et al., 1996) and magnesium with oxalic acid (Gold et al., 1997). These authors showed that iron removal reduced radical release, and accordingly reduced DNA and lipid damage.

Although the physical aspect ratio of asbestos fibres may affect carcinogenicity, research has identified other factors affecting the toxicity of asbestos fibres in mammals, including the positive charge on the surface of the chrysotile structure (Holmes and Lavkulich, 2014). Monchaux et al. (1981) found dramatically higher incidences of mesothelioma in rats injected with untreated chrysotile compared with those injected with fibres treated with hydrochloric or oxalic acid. Nevertheless, targeting the removal of cations (predominantly iron or magnesium) appears to be a key factor to reducing toxicity, and the use of bioremediation to reduce free metal concentration from the surface of the fibres may have great potential. Further breakdown of the fibre structure by the action of secreted organic acids may over time completely dissolve fibres, subject to unknown timescales. The acute inflammatory response from injecting asbestos into the peritoneal space of mice apparently contributes to MM pathogenesis via the repeated release of chemokines such as IL-1b, IL-6 and TNFa (Kadariya et al., 2016; Pietrofesa et al., 2016). Such mouse studies coincide with other experimental studies documenting that asbestos induces cell inflammation connected with the initiation and progression of MM. The Nalp3 inflammasome (Dostert et al., 2008) and high-mobility group box 1 (HMGB1) protein (Yang and Tracey, 2010) have been identified as key initiators of this proinflammatory process.

## CASE STUDIES

The following three case studies demonstrate some scenarios in which ACM waste may create public health issues; the current lack of viable, cost-effective alternatives to disposal in hazardous-waste landfills; and the need for long-term disposal strategies for these wastes, especially for countries with limited landfill facilities. Case studies were selected to cover a wide variety of climatic zones and also due to the geographical area of the expertise of the a liated key researchers.

### Case Study 1 – Mr Fluffy Asbestos Insulation in Australia (University of Canberra)

This case study came about as part of the major research project being led by Unitec Institute of Technology, and a visit to Canberra, Australia by two of the researchers. Their visit coincided with the peak of the asbestos removal project being undertaken by the ACT Government. The University of Canberra, Faculty of Arts and Design have provided full support of the overall project and the case study. The illustrations are courtesy of a site visit to a removal operation.

Australia was a high user of asbestos until the early 1990’s and, both crocidolite and chrysotile were mined there (Australian Government.Asbestos Safety and Eradication Agency [ASEA], n.d.; Department of Planning and Environment, n.d.; Environmental Health Standing Committee, 2013). Asbestos products were also imported until a broad ban was introduced in 2003 (Environmental Health Standing Committee, 2013). Now, asbestos is considered likely to be in some building material in three of every four homes in Australia built prior to the mid-1990s (Australian Government.Asbestos Safety and Eradication Agency [ASEA], n.d.). This poses a continuing national health risk, enhanced by a growing trend for home renovation by inexperienced and untrained homeowners (Gordon and Leigh, 2011).

**FIGURE 4 |:**
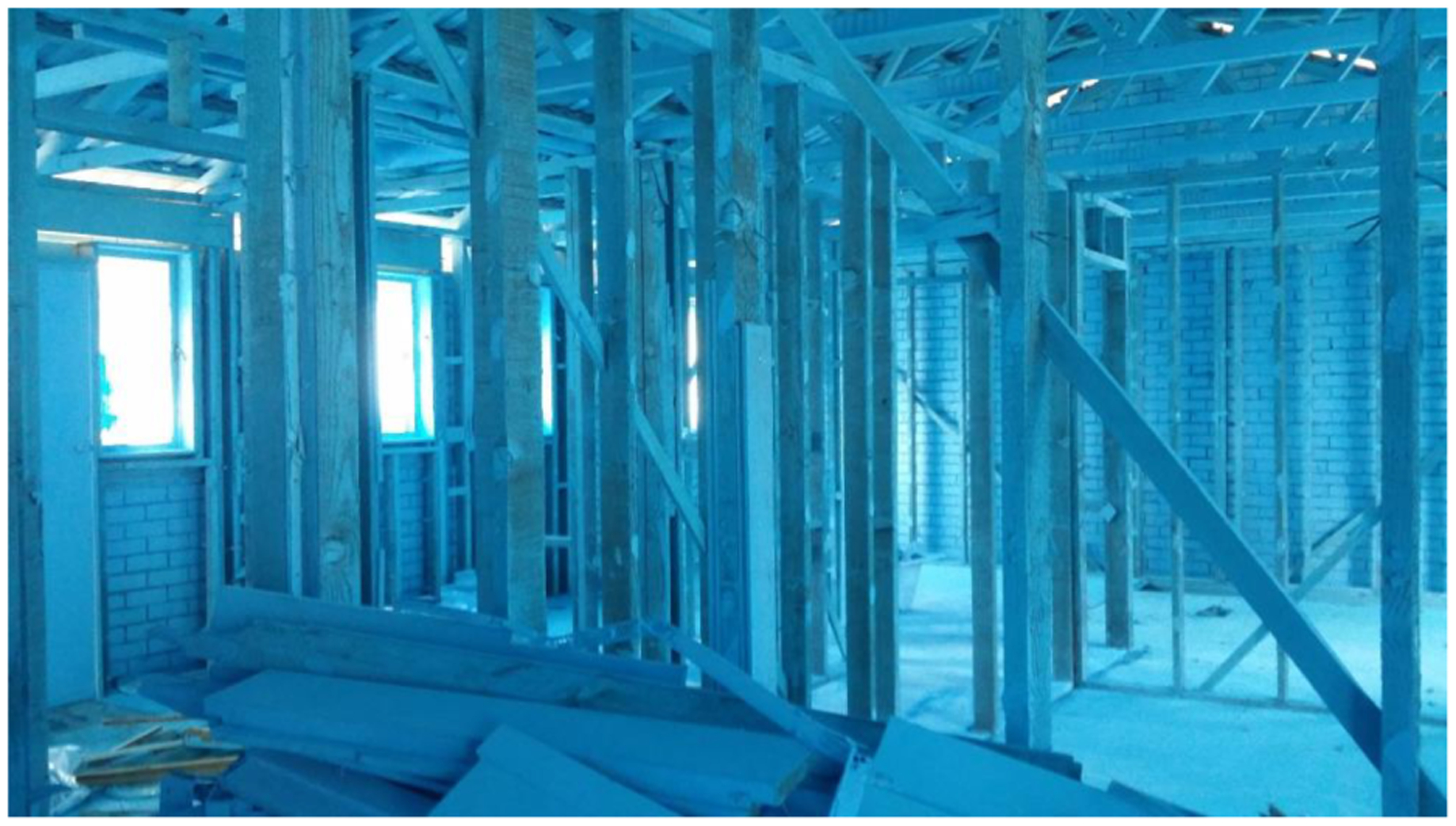
A decontaminated Mr Fluffy home sprayed with a mixture of PVA and blue paint to bind any remaining loose fibres prior to removal to landfill.

Homes insulated with ‘Mr Fluffy’ loose asbestos fibres are an example of this legacy (Figure 4) where in the 1960s and 70s, crushed asbestos was installed as insulation in some homes and buildings. Despite warnings about the potential dangers, the loose-fill product was pumped into about 1,100 residential properties in Canberra and the adjoining state of NSW (Australian Capital Territory Canberra [ACT] and Auditor-General, 2016). By 1983, following reports and warnings, the Commonwealth Government prohibited use in its properties, and began to remove asbestos from any Commonwealth-owned buildings (Australian Capital Territory Canberra [ACT] and Auditor-General, 2016). The removal programme was extended to about 65,000 homes in 1988 and completed by 1993 (Australian Capital Territory Canberra [ACT] and Auditor-General, 2016).

Little more was done until 2011, when owners of a Mr Fluffy-insulated house discovered that it had not been remediated in the original removal programme and had significant contamination (Australian Capital Territory Canberra [ACT] and Auditor-General, 2016). With financial assistance from the Commonwealth Government, a local government taskforce was established, and a programme began in 2014 to purchase and demolish 1,022 affected homes in the ACT at an expected cost of about $US750 million (Australian Capital Territory Canberra [ACT] and Auditor-General, 2016). The net total cost (acquisition plus demolition less subsequent sale of land) with prudent management by the government is currently estimated at approximately $US235 million (The Legislative Assembly for the Australian Capital Territory, 2017). Extracted loose fibres from the homes are sealed in thick plastic and buried at a special site marked by global positioning satellite (GPS). The remaining ACM is then sprayed with a mixture of polyvinyl acetate (PVA) and paint prior to demolition and then hauled, along with any contaminated soil, to a separate landfill and buried. On completion of demolition to all affected homes, this landfill site will be remediated and used as parkland.

This case study shows that even with a well-funded and sophisticated decontamination program, large quantities of asbestos-contaminated soil remain which need to be stored in hazardous landfills for the long-term. This is an on-going hazard which could re-emerge due to potential future pressure to amend the land use of the site for land development and possible natural land movement processes.

### Case Study 2 – Asbestos-Contaminated Land in the Cook Islands (Unitec Institute of Technology)

This case study was carried out as part fulfilment of a final year student project by Unitec Institute of Technology supervised by Berry (Unitec Institute of Technology) and in collaboration with K2 Environmental Ltd (Rarotonga).

There has been prolific use of asbestos-containing materials in the Pacific Islands. A recent survey identified that approximately 3% of houses and public buildings contain asbestos (Secretariat of the Pacific Regional Environmental Programme [SPREP], 2015), mainly ACM construction products. A particular problem is “Super Six” roofing (Secretariat of the Pacific Regional Environmental Programme [SPREP], 2011), which becomes brittle and releases asbestos fibres with weathering (Bowler and Caughnley, 2014).

Asbestos was uncovered in reconstructing two of ten schools in Rarotonga, the largest of the Cook Islands. The topsoil surrounding the main building of each was contaminated with high levels of asbestos (K2 Environmental Ltd., 2014) from wall cladding and from Super Six roofing product that previously covered all classrooms.

Four options were proposed to deal with contaminated topsoil. These complied with New Zealand legislation and best practice (Berry and Wairepo, 2015):

1. Capping (sealing, enclosing or encapsulating) internal walls and external soil
2. Removal and disposal at a local landfill
3. Removal and disposal at an international landfill
4. Removal and disposal at sea

Of the four options, initially capping was the least preferred option, based mainly on cost but also local preference. However, for local removal and disposal, a significant upgrade of the landfill facilities would have been required, including lining and covering the waste material. For international disposal, there was potential for long-term storage in containers for later removal to a specialised waste disposal unit overseas; however, strict quarantine regulations (in New Zealand and Australia) combined with high costs made this infeasible.

Despite public opposition, the eventual solution at both schools was on-site burial within school grounds (3 m deep with 200 μm polythene covering), with cost as the main determinant. The removal and disposal of asbestos at the two schools was estimated at US$250,000. The low cost option of on-site waste detention outranked alternative long-term sustainable options (described above). A key finding of this study was the recommendation for larger countries, with a greater capacity for both treatment and disposal, to consider foreign aid on a case-by-case basis (Berry and Wairepo, 2015). These schools represent a small fraction of the Pacific Island buildings believed to contain asbestos. The predicted cost of the removal of all the asbestos-contaminated materials in the Pacific Islands is US$110m (Williams, 2015).

This case study provides an example of an island nation without local hazardous landfill capacity and is highly restricted in its capability to export wastes. Therefore, large amounts of asbestos contaminated material and soils cannot be addressed appropriately with the current options available.

### Case Study 3 – Asbestos Waste in Ambler, Pennsylvania, United States (University of Pennsylvania)

Ambler is a suburb near Philadelphia, Pennsylvania and was investigated by Emmett (University of Pennsylvania). Information collection and processing for the case study of Ambler was approved by the Institutional Review Board (IRB) of the University of Pennsylvania.

Ambler (population 6,500 in 2014), was for many years the site of the world’s largest manufacturer and supplier of chrysotile asbestos-containing products, particularly from the 1910s to the 1930s, production continued until the 1980’s. A local cluster of MM cases in Ambler was confirmed in 2011 (Pennsylvania Department of Health, 2011), which was thought to be related to past exposure to airborne asbestos fibres.

**FIGURE 5 |:**
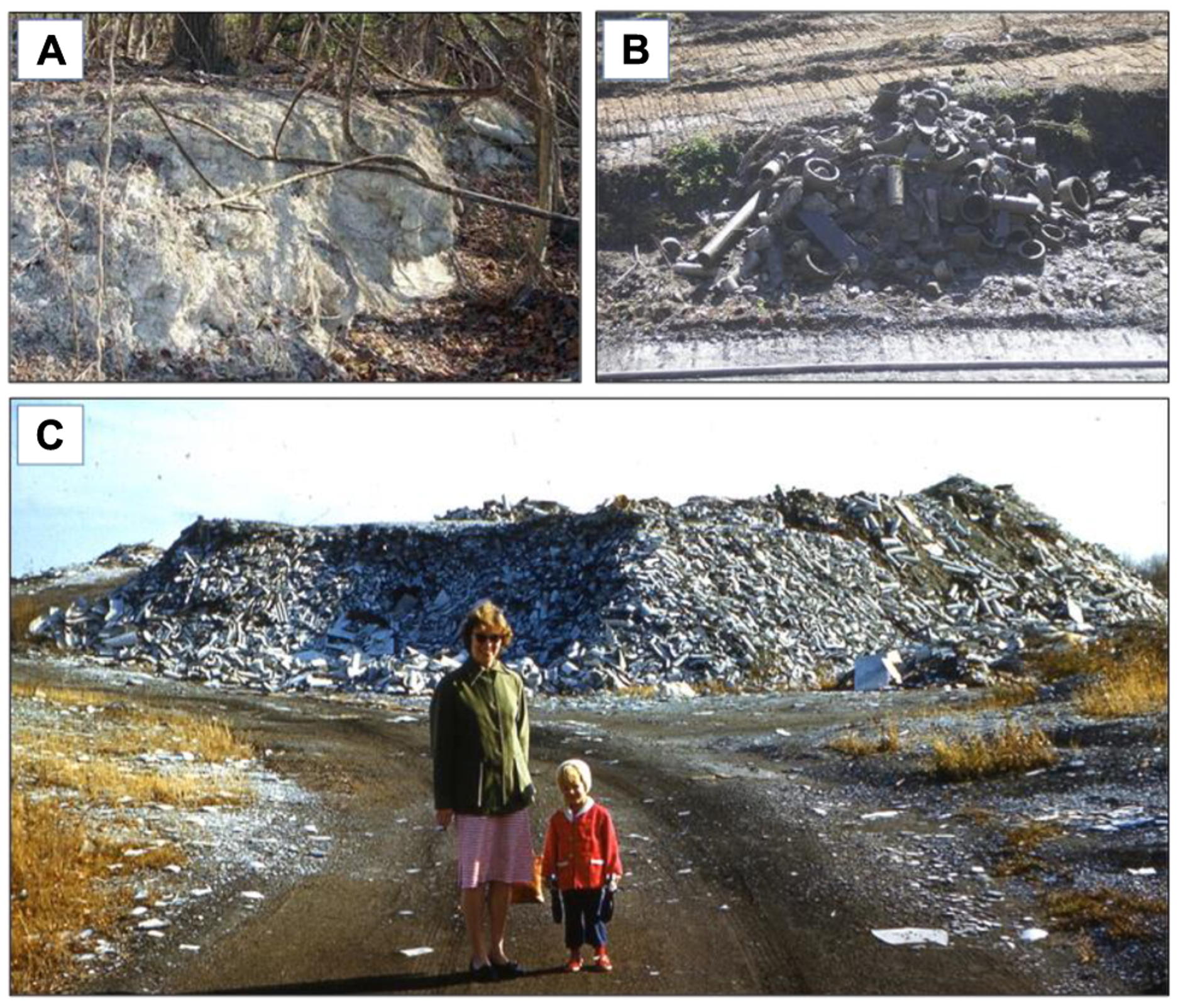
Asbestos fibres in Ambler, PA. (**A**) Ambler ‘snow’ – asbestos uncovered by vegetation removal at Ambler Piles. (**B**) Piles of various asbestos contaminated waste from Rose Valley Creek Banks in Ambler. (**C**) The Bo-Rit ‘Asbestos Mountain’ circa 1960. Photographer: Joe Marincola (with permission from Greg Marincola); reproduced with permission from Springer, Inc. (Emmett and Cakouros, 2017; p. 116).

Ambler’s ACM waste contamination in an urban setting has required extensive remediation (Figure 5). Large amounts of ACM waste existed in two locations called the Ambler Piles and Bo-Rit. The Ambler Piles or “White Mountains” of Ambler (Figure 5) adjoins a residential area. It was a 10 ha, 9 m high asbestos-containing waste area, with some areas up to 21 m above natural grade. When it was added to the U.S. EPA National Priorities List (NPL) as a “Superfund” site in 1984, it was estimated then to contain more than 750,000 m^3^ of ACM.

In an emergency response to reduce the hazard in 1984, the EPA covered exposed areas with soil, stabilised slopes, hydro-seeded, installed a drainage system, dismantled an adjacent playground and fenced off the area with warning signage (United States Environmental Protection Agence [EPA], 1988). The remediation that followed involved covering the area with soil and geotextile fabric, grading with soil and additional fencing (United States Environmental Protection Agence [EPA], 1989); this was completed in 1996. The area continues to be monitored through 5-year reviews and annual visual inspections by EPA. Visible waste products (presumably ACMs) are brought to the surface by erosion, the roots of fallen trees or animal burrowing. Unlike Case Study 2 (Cook Islands), this remediation did not include polythene covering and the resulting remediated area was not designed for immediate use by civilians. However, despite perimeter fencing, there is evidence the area is still accessed.

In contrast, the second site, Bo-Rit (approximately 11 ha), underwent a state-of-the-art ‘cap-in-place’ remediation. It is also adjacent to residential housing. Following identification of asbestos in the 1980s, warning signs and fences were erected at the site and adjacent playing fields (AGES, 1984). EPA inspection in 1987 found up to 22% asbestos in soil samples, and the site was added to the NPL in 2009.

Bo-Rit consists of three distinct adjacent land parcels: an asbestos pile, a reservoir parcel and a park parcel. The 2.4 ha pile of asbestos-product-manufacturing waste was elevated up to 6–9 m above its surroundings, 12 m deep and uncapped (Figure 5). It had been vegetated with native herbaceous flora since 1965 and fenced since 1986. The 6 ha reservoir parcel contained a man-made reservoir with a berm constructed of asbestos shingles, millboard and soil. In later years it was a waterfowl reserve. The 4.5 ha park parcel housed an estimated 149,000 m^3^ of out-of-specification asbestos products and other solid waste, distributed across the park, up to 4 m deep, with an average cover of 0.24 m of soil. The area had been used as a public park from at least 1973 until it was officially closed to the public and fenced in 1984 (CDM Federal Programs Corporation [CDMSmith], 2013). A local creek and two tributaries flow adjacent to or through the three parcels and are subject to periodic flooding. Areas of the Bo-Rit site are within the 100-year flood plain (CDM Federal Programs Corporation [CDMSmith], 2015).

Work to remove the threat to health primarily by capping in-place was performed from 2008 to 2017. ACM and trees were removed from the banks and beds of the waterways crossing the site to prevent disturbance of underlying ACM should trees fall. The slopes of the asbestos piles were smoothed to a gentle gradient before the area was covered with geotextile fabric topped with geo-cells, a honeycomb-like web filled with soil to a depth of at least two feet and seeded. Banks were covered with loose stones to prevent erosion. The reservoir was drained, its floor and sides covered, and its ACM berm reinforced and covered with clean soil. All work was constantly wet-sprayed to prevent asbestos becoming airborne. Onsite, stationary monitors and personal monitors worn by workers were used to ensure rapid detection of any airborne asbestos. Severe flooding during operations damaged the site and resulted in a number of modifications, including widening the creek, and installing cement cables to anchor rip-rap and a floodgate (United States Environmental Protection Agence [EPA], 2016a). Since then, no serious flooding of the site has been reported. The remediation appears visibly superior to the previous work on the Ambler site.

This ‘cap-in-place’ remediation cost US$27.1m (estimated present-day value). This was much cheaper than other evaluated alternatives: excavation and off-site disposal (US$269m), *in situ* joule heating (US$257m), and excavation, on-site *ex situ* Thermo-Chemical Conversion Treatment (TCCT) and on-site disposal (US$267m). Other options were dismissed as infeasible or prohibitively expensive (United States Environmental Protection Agence [EPA], 2016b).

Now that the remediation is complete, the site will be available for limited public use. Tentative planning is currently underway with community input for uses that include a waterfowl reserve and reopening of the park for recreation as part of an extended green strip along the Wissahickon Creek (United States Environmental Protection Agence [EPA], 2016a).

This case study illustrates that capping and a suitable vegetation cover is the remediation method of choice, with a huge cost advantage over other options, although not the best option for bioremediation, which is explained below. It also illustrates the inadequacy of fencing and signage to prevent human access, the importance of erosion control and other controls at the site, as well as the need to prepare sites for extreme weather events.

The example of the Ambler super fund site highlights that industrial contamination of land can lead to large areas which cannot be decontaminated. In this instance, containment *in situ* via capping provides an option which though long-term requires constant management to prevent release due to erosion or human interference.

The three case studies demonstrate how long term disposal options for asbestos contaminated soils remain an unsolved problem. In combination with the current uncertainties around the pathways for asbestos mobility in soils (discussed further in see section “Asbestos Mobility in Soils”), this indicates a need to investigate long-term solutions to treat asbestos contaminated soils. Bioremediation may provide a novel long term solution which is low energy, has potential for scale-up for large areas and availability for countries without sophisticated waste management systems.

## ASBESTOS MOBILITY IN SOILS

Air transport is the exposure pathway of concern for asbestos. Consequently, its containment at waste sites usually involves capping ACM to inhibit wind spread as demonstrated by the case studies. However, do buried asbestos particles remain in place? An understanding of how water and substrate chemistry control asbestos mobility in soil will allow us to manipulate variables to immobilize or remove asbestos at waste removal sites.

Asbestos fibre transport through soil by groundwater flow has been considered negligible (United States Environmental Protection Agence [EPA], 1978). However, there is accumulating evidence that asbestos particles may travel long distances within aquifers (Kashansky and Slyshkina, 2002; Koumantakis et al., 2009; Buck et al., 2013; Schreier and Lavkulich, 2015; Turci et al., 2016). Situations that allow or enhance the mobility of asbestos in groundwater – for example, shallow groundwater interacting with the soil surface, or disturbance of the subsurface soil – may result in the escape of fibres from capped landfills to the soil surface, where they may become airborne.

What do we know about the mobility of asbestos? The mechanisms that govern the transport potential of asbestos particles in groundwater are just beginning to be explored, and new understanding will be vital to future waste containment strategies.

Like any other colloid, the mobility of asbestos fibres depends on particle size and shape, and the soil medium’s pore size distribution. Colloid size can significantly influence settling (Semmler et al., 2000; Auset and Keller, 2004) and electrostatic effects (attraction-repulsion) (Zhuang et al., 2005). Larger colloids are more likely to be trapped in narrow soil pore throats (straining), or to collide with and be retained at the solid-water interface (McDowell-Boyer et al., 1986). Colloid shape can also influence straining and attachment in soil (Ryan and Elimelech, 1996; Wang et al., 2012), and the shape of colloids has more effect on whether colloids diffuse in water or form aggregates than do their material properties (Wu et al., 2015, 2017). Rod-like colloids attach to the solid-water interface at a higher rate than spherical colloids, due to surface heterogeneity (Seymour et al., 2013). Although the elongated shape and large specific surface area increase the straining and attachment of asbestos fibres in soil media, dissolved organic carbon (DOC) could enhance their mobility and transport as it does other contaminants (McCarthy and Zachara, 1989; Beesley et al., 2010).

Although loose, well-dispersed asbestos fibres may move large distances in the environment under favourable conditions, their mobility in general is hindered if they form aggregates. Colloidal aggregate formation and attachment to soil is controlled by chemical factors such as the water’s pH and ionic strength, through changing the effective surface charge of colloids and soil (Bradford et al., 2002; Wu et al., 2017).

We need further understanding of the controls of water and substrate chemistry on asbestos mobility in soil to be able to either immobilize asbestos by enhancing attachment *in situ*, or to remove asbestos from contaminated soils by detachment and flushing.

## TREATMENT OPTIONS

The transformation of asbestos into non-toxic products has been attempted before, with several positive outcomes. But there are disadvantages in most cases, usually due to high energy requirements or health and safety issues. Spasiano and Pirozzi (2017) discussed physico-chemical treatment options in detail and concluded there are many barriers to overcome before an acceptable alternative to landfilling is devised. Whilst landfill costs are increasing significantly as land availability becomes an issue, the current low costs of landfill disposal mean that it is unlikely that these types of treatment will be economically viable in the foreseeable future for large ACM aggregations.

### Bioremediation of Asbestos Fibres

Biological treatment processes for the degradation of asbestos fibres have been largely overlooked due mainly to the inorganic nature of the asbestos structure and the unknown (and predicted long) timescales. The following sections describe these processes, and outline evidence suggesting that they can successfully reduce the toxicity of asbestos.

Asbestos may be degraded to some extent by the action of biological organisms, particularly soil microorganisms, including some fungi, and also lichens. Although all organisms require organic substances as growth substrates, inorganic substances may provide energy for microbial metabolism, and may also supply essential trace elements, such as iron. The removal of iron from asbestos by organisms could reduce its carcinogenic potential.

Bioremediation of asbestos-contaminated sites could enlist any of several processes, depending on the type and concentration of the asbestos mineral, the scale of the site, and the presence of other contaminants, such as toxic heavy metals (Cunningham et al., 1995; Favas et al., 2014; Prasad, 2015; Prasad and Shih, 2016). Possibilities include phytostabilization, where plants are used to stabilise the substrate but not necessarily alter the asbestos (Kumar and Maiti, 2015; Kumar et al., 2017); phytoextraction if plants can hyper-accumulate certain elements in sufficient quantities that harvesting plants will reduce cooccurring soil metal contaminants (Sheoran et al., 2009; van der Ent et al., 2015); and rhizodegradation or bioweathering, where plants and/or soil microbes chemically alter asbestos fibres (Daghino et al., 2006, 2009; Favero-Longo et al., 2013).

### Phytostabilization

Introducing new plant cover can be especially useful when abandoned asbestos mines or contaminated sites are too large to be contained by a manmade cap topped with uncontaminated soil and vegetation (United States Environmental Protection Agence [EPA], 2015). Most asbestos mines or disposal sites present no or little vegetation cover, mainly due to severe infertility (Meyer, 1980). Plants need to be able to tolerate the conditions of the underlying substrate (Favero-Longo et al., 2006). The choice of plant species and/or soil amendment requires evaluation of soil pH, heavy metal presence, nutrient levels and local soil and climatic conditions. Plant cover quickly reduces soil erosion and limits asbestos fibre dispersion (Liston and Balkwill, 1997). However, protection must be provided against the periodic exposure of fibres through, for example, landslides or animal burrows.

Some plants and lichens spontaneously develop on asbestos-rich substrates, and mature plant communities can then colonise the debris, completely covering it (Favero-Longo et al., 2006). Favero-Longo et al. (2006) found that lichen species can colonize asbestos veins in serpentinite rocks, creating a natural “cap,” and thereby potentially reducing air dispersion of fibres. Some initial research has been done on artificially establishing lichens in an old asbestos mine (Favero-Longo and Piervitorri, 2012).

### Bioweathering

Bioweathering is the biologically induced or aided breakdown (‘weathering’) of rocks or mineral-based substances and can occur in hot and cold, sub-tropical, tropical and temperate climates (Hirsch et al., 1995). Its mechanisms are not fully understood, and there is a paucity of data regarding degradation rates. It has been demonstrated that fungi and lichens can remove iron from solid asbestos materials. This section describes how bioweathering occurs, and the evidence for its effectiveness with asbestos.

Biological soil communities that degrade rock include fungi, lichens, and free-living cyanobacteria and algae (Hirsch et al., 1995; Balloi et al., 2010). These organisms can dissolve mineral substrates, and use minerals as an energy source, as a key part of respiration, or, according to Ehrlich (1996), to satisfy a trace metal requirement. Erlich observed that microbes ‘may satisfy some or all of their trace element requirements and that of other organisms in their community with dissolved mineral constituents.’

Geochemically, lichens produce weathering via three main known processes. First, lichens’ respiratory CO_2_ dissolves in water in the thallus, producing carbonic acid (Adamo and Violante, 2000; Chen et al., 2000). Second, lichen fungus synthesizes oxalic acid, producing oxalates from the minerals within the rock (Adamo and Violante, 2000; Chen et al., 2000). Third, some of lichens’ secondary metabolites produce soluble metal cation-organic complexes when in contact with minerals (Adamo and Violante, 2000; Chen et al., 2000). Physically, hyphae can penetrate rock spaces, and thalli expand and contract with wetting and drying. Lichen substances can mechanically disrupt the rock matrix when they crystallize, and the mineral particles of rocks can become incorporated into the thallus (Adamo and Violante, 2000; Chen et al., 2000). The hyphae of lichens have been shown to penetrate *>*2 mm into chrysotile, surrounding individual fibres (Favero-Longo et al., 2005). Lichen and fungal species have been shown to selectively deplete cations from chrysotile, degrading the fibres, and extracting magnesium and iron (Favero-Longo et al., 2005, 2007; Daghino et al., 2006).

In laboratory studies, the surface properties of chrysotile asbestos have been reportedly changed by organic and inorganic acids, simulating the weathering process (Holmes and Lavkulich, 2014), with oxalic acid being most effective at extracting the majority of trace elements present in the chrysotile. The acid reduced the positive charge and produced visible changes at the fibre surface (Holmes and Lavkulich, 2014). The chelating activity of lichen metabolites has been linked to a decreased chemical reactivity of chrysotile (Favero-Longo et al., 2009). Lichen growth *in situ* on asbestos-contaminated material stimulated partial incongruent dissolution of chrysotile and crocidolite fibres, which reduced surface reactivity (Favero-Longo et al., 2009).

Fungi can also penetrate deeply into cracks and cavities, aided by biochemical dissolution of parts of the rock matrix. Some fungi produce iron-chelating siderophores, and/or organic acids which lower the pH and form chemical complexes with some mineral components of rocks, including different forms of asbestos (Daghino et al., 2006, 2008; Mohanty et al., 2018). For example, Daghino et al. (2005) reported that *Fusarium oxysporum* extracted iron from crocidolite, amosite and chrysotile, and Daghino et al. (2008) found that some fungal species isolated from two chrysotile mines removed iron from the chrysotile fibres using siderophores.

Bioremediation of asbestos by fungi, particularly *Fusarium oxysporum* and *Verticillium leptobactrum*, has been tested in controlled laboratory studies. These two species have been repeatedly isolated from naturally occurring serpentinic rocks that contain asbestos particles, suggesting that they adapt easily to this selective mineral substrate (Martino et al., 2004; Daghino et al., 2005).

Experiments have shown that the chelating activity of exudates from some fungi and lichen (which has a fungal component) modify the chemical composition of chrysotile fibres *in vitro*, affecting their chemical reactivity and structure and potentially altering toxicity. These organism-driven weathering processes can reduce chrysotile fibre toxicity (Daghino et al., 2006, 2009), and accordingly increase iron (Fe), magnesium (Mg) and nickel (Ni) concentrations in surrounding substrates (Chardot-Jacques et al., 2013). These dissolved elements could provide plant nutrition but can also be lost though leachate (Chardot-Jacques et al., 2013). In one experimental study, the iron released was not incorporated into the fungal biomass (Daghino et al., 2008), but the fungi’s progressive removal of reactive iron ions, which are responsible for asbestos’s DNA damage, was encouraging (Daghino et al., 2006).

More recently, Mohanty et al. (2018) performed experiments on crushed chrysotile fibres with environmentally realistic concentrations of three different organic acids and fungal and bacterial siderophores. They found that both the bacterial and fungal siderophores significantly removed iron from chrysotile, but the organic acids did not. The siderophores were effective within the fibre as well as the surface layers. They suggested that the high pH of some asbestos serpentine soils would limit the iron-chelation efficacy of organic acids.

These studies have been trialled on pure asbestos materials; it is less clear how effective these iron-removing processes would be where asbestos fibres are dispersed in soil. Loose fibres have a more exposed surface area, which could increase the rate of chelation by fungi and lichens, but this possibility needs to be investigated. The case studies discussed previously relied on landfill disposal or *in situ* capping, neither of which are likely to produce the microbial communities required for bioremediation. This is especially true given that simply providing these communities in isolation from other key biological processes may not be enough to support fibre degradation. The inclusion of plants and trees within the treatment area may be required.

### The Role of Roots

Microbial activities in the rhizosphere (mainly due to bacteria and fungi) can alter the chemical and physical properties of surrounding soil. Although a rhizosphere extends only a few millimetres from the root surface, the total root length can be immense (estimated at a staggering 70,000 m for a single wheat plant) (Bolton and Fredrickson, 1993). This area often hosts mycorrhizal fungi, which establish symbiotic associations with the roots of most plant species (Bolton and Fredrickson, 1993) and can supply inorganic nutrients to their plant host (Girlanda and Perotto, 2005). Other rhizospheric fungi that do not form mycorrhizal associations, such as saprotrophic fungi, may act as biofertilizers through rock weathering – mobilising essential plant nutrients directly from minerals (Hoffland et al., 2004).

Processes in the subsoil may support microbial activity to alter the structure of asbestos fibres. For example, living roots can release carbon compounds in a process known as rhizodeposition. This process is essential to the development of a complex microbial community at the root-soil interface because it provides both a nutrient source and stimuli for growth and metabolic activities (Girlanda and Perotto, 2005). In addition, tree roots may selectively support certain soil microbes, including mycorrhizae that contribute to nutrient mobilisation, thereby providing nutrition for the trees (Calvaruso et al., 2010).

It is likely that a plant’s nutritional requirement for iron and other trace metals can be symbiotically satisfied by microbial action working on asbestos fibres, thus reducing their carcinogenicity. This process could potentially be sped up by applying seed bacteria or fungi from serpentine soils or mining waste. The release of siderophores, organic acids and/or melanins responsible for iron solubilisation may be linked to a lack of bioavailable iron in the soil (Haas, 2003; Ghaderian et al., 2007). This suggests that a continual iron sink drives the process, and the role of bacteria in acquiring trace amounts iron for plants is well known (Borin et al., 2010).

## PROPOSED ASBESTOS BIOREMEDIATION SYSTEM

**FIGURE 6 |:**
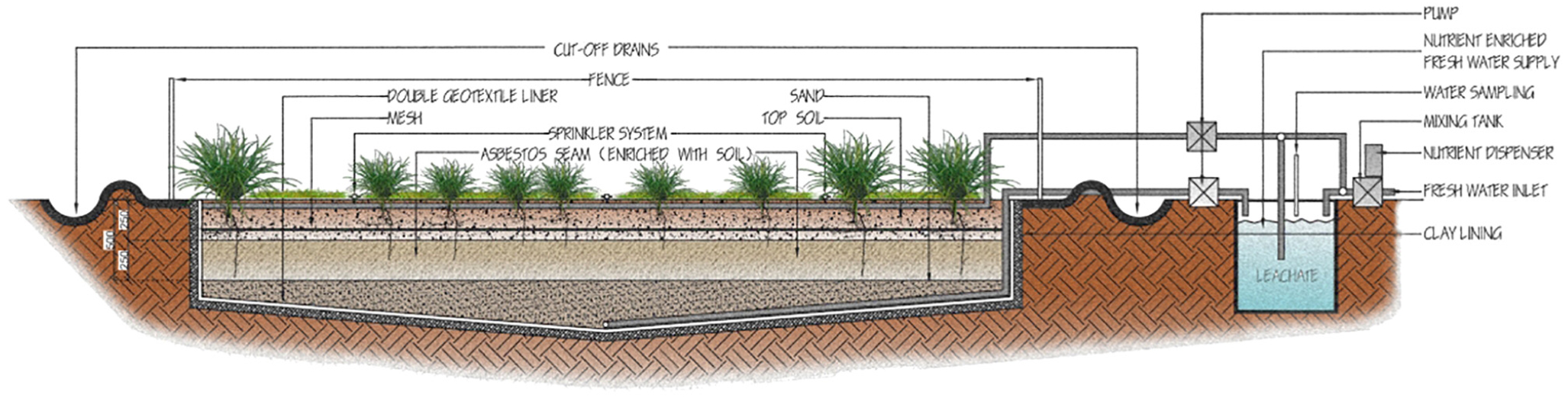
Activated landfill for testing the potential for the bioremediation of asbestos and asbestos contaminated waste.

We are a consortium of researchers from three different countries proposing to test the potential of bioremediation to reduce the toxicity of asbestos-containing waste. Above such waste, at multiple sites, we will create pilot-scale controlled ecosystems that include bacteria, fungi and plants. The system will become an ‘activated landfill’ (Figure 6). We will test whether the organisms’ natural biological activities can remove a crucial element of asbestos’s carcinogenic action: iron.

**FIGURE 7 |:**
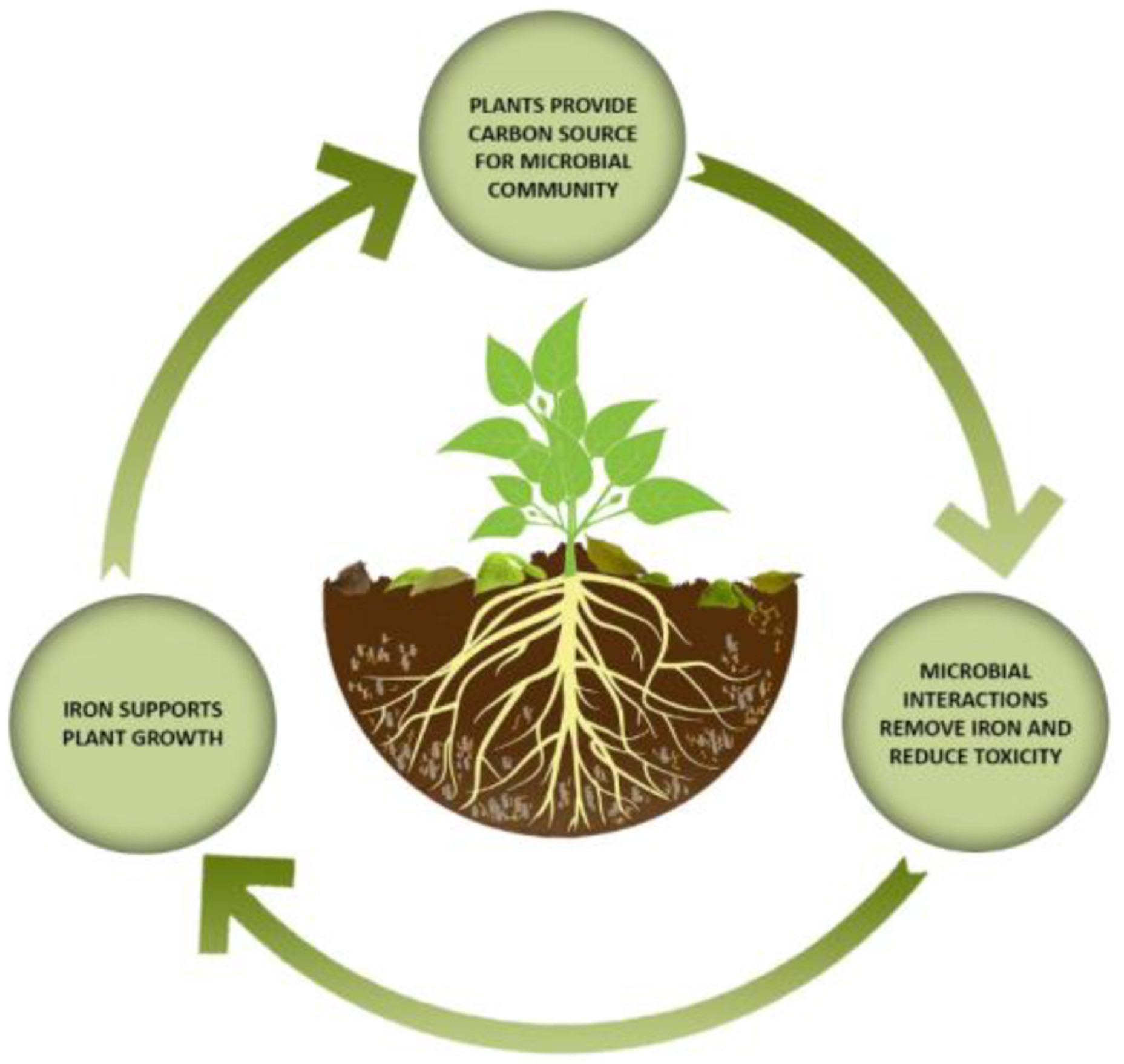
The importance of the symbiosis between bacteria, fungi, and plants.

A complete ecosystem is almost certainly crucial, since symbiosis between species may provide both the catalyst and the driver for continuous fibre degradation. The ability of microorganisms to metabolise and grow in a wide range of different environments is usually a result of interactions with other members of the community (Hirsch et al., 1995). Conversely, bioweathering of asbestos fibres by lichen-forming ascomycetes has been strongly limited by time and the absence of wider biological communities and related symbioses (Favero-Longo et al., 2007). We believe that a system comprising microbes and plants may also serve to protect public health during bioremediation processes. In the following discussion, we link the anticipated role of the various biotic groups in a controlled ecosystem, as summarised in Figure 7.

The questions we aim to answer from our planned research include: what is the rate of bioweathering in the field for asbestos fibres, how can one measure variation in degradation rates, and why does this variation occur? For example, is this driven by climatic conditions or variations in microbial diversity? How mobile are the asbestos fibres in different soil types? Is a degree of phytoremediation feasible and, if so, which types of (native) vegetation will support this?

We will be investigating factors that affect bioremediation efficiency: the nature of the contaminant, mass transfer within the soil profile and availability of degrading soil microflora. The activity of the microorganisms also depends on many factors: contaminant uptake and bioavailability, concentration, toxicity, mobility, access to other nutrients and activated enzymes, and possibly others. Choosing suitable plants will be paramount. We will investigate the ecology of both the planned remediation sites and uncontaminated neighbouring sites, which will provide valuable information on the types of plant communities capable of developing. Drastically disturbed ecosystems can take a long time to reach equilibrium. Vegetation with high productivity and decomposable litter provide a favourable environment for microorganisms. It is essential that surface vegetation such as trees and shrubs should not bring up the contaminants for example, through windfall (Nicholson and Safaya, 1993).

Timescales for the reduction of carcinogenic potential are difficult to predict, although substantial lichen and plant growth has developed naturally after 35–45 years in abandoned asbestos mines. This colonisation was hindered by stability, morphology and microclimate of the rocks rather than the asbestos. This suggests that the timescales required for a more managed approach will be much less; for example, the underlying substrate will not be purely mineral, but will be enriched with organic material to boost microbial growth and add nutrients. Initial trials will be planned for 5-year periods.

Any experimental setup will be insulated from the groundwater, as the mobility of asbestos fibres, especially once partially biodegraded, is not yet well enough known. Leachate will be prevented from entering groundwater by a barrier, and it will be recycled, which will also allow easy access for sampling to test for mobilized asbestos fibres. A mesh close to the surface will prevent larger animals from burrowing into the asbestos layer, while still allowing water and roots to access the asbestos bioremediation layer.

## CONCLUSION

We propose that the way we deal with waste, and in particular hazardous asbestos waste, can be improved upon. Packaging asbestos waste in plastic, covering with soil and sealing it is not a magic trick, and leaves a toxic legacy on a huge scale for future generations.

Nature can convert highly hazardous substances into potentially less hazardous forms, and current waste management practices actively prevent this from happening. Evidence suggests that a combination of microbial weathering and phytoremediation could provide a process that at least partially remediates asbestos fibres and asbestos-contaminated materials. The perpetual iron sink provided by plant growth could trigger microbes to persistently release chelating molecules, which would reduce the iron that makes asbestos fibres carcinogenic. Bioremediation techniques have many benefits including low energy consumption, large scale-up potential, ecosystem conservation and suitability for locations without sophisticated waste management facilities. Using bioremediation for inorganic substances (such as asbestos) is a novel approach which may be expanded to include other asbestiform minerals of concern, such as erionite.

To remediate asbestos-containing waste, we plan to create a conducive environment for an ecosystem including bacteria, fungi and plants. We hope to learn how to create optimal conditions for this process to proceed over short, beneficial timescales.

## Figures and Tables

**TABLE 1 |:** Global contamination limits for asbestos in soils.

	New Zealand (Hillman and Corbett, 2014)	Australia (Government of Western Australia Department of Health, 2009)	Netherlands (Hillman and Corbett, 2014)
Bonded	100 mg/kg	100–500 mg/kg	100 mg/kg
Friable	10 mg/kg	10 mg/kg	100 mg/kg
